# Perspectives and Experiences With Large Language Models in Health Care: Survey Study

**DOI:** 10.2196/67383

**Published:** 2025-05-01

**Authors:** Jennifer Sumner, Yuchen Wang, Si Ying Tan, Emily Hwee Hoon Chew, Alexander Wenjun Yip

**Affiliations:** 1 Alexandra Research Centre for Healthcare in a Virtual Environment Alexandra Hospital Singapore Singapore; 2 School of Computing National University of Singapore Singapore Singapore

**Keywords:** digital health, artificial intelligence, survey research, large language model, healthcare, survey, workforce, healthcare worker, professional

## Abstract

**Background:**

Large language models (LLMs) are transforming how data is used, including within the health care sector. However, frameworks including the Unified Theory of Acceptance and Use of Technology highlight the importance of understanding the factors that influence technology use for successful implementation.

**Objective:**

This study aimed to (1) investigate users’ uptake, perceptions, and experiences regarding LLMs in health care and (2) contextualize survey responses by demographics and professional profiles.

**Methods:**

An electronic survey was administered to elicit stakeholder perspectives of LLMs (health care providers and support functions), their experiences with LLMs, and their potential impact on functional roles. Survey domains included: demographics (6 questions), user experiences of LLMs (8 questions), motivations for using LLMs (6 questions), and perceived impact on functional roles (4 questions). The survey was launched electronically, targeting health care providers or support staff, health care students, and academics in health-related fields. Respondents were adults (>18 years) aware of LLMs.

**Results:**

Responses were received from 1083 individuals, of which 845 were analyzable. Of the 845 respondents, 221 had yet to use an LLM. Nonusers were more likely to be health care workers (*P*<.001), older (*P*<.001), and female (*P*<.01). Users primarily adopted LLMs for speed, convenience, and productivity. While 75% (470/624) agreed that the user experience was positive, 46% (294/624) found the generated content unhelpful. Regression analysis showed that the experience with LLMs is more likely to be positive if the user is male (odds ratio [OR] 1.62, CI 1.06-2.48), and increasing age was associated with a reduced likelihood of reporting LLM output as useful (OR 0.98, CI 0.96-0.99). Nonusers compared to LLM users were less likely to report LLMs meeting unmet needs (45%, 99/221 vs 65%, 407/624; OR 0.48, CI 0.35-0.65), and males were more likely to report that LLMs do address unmet needs (OR 1.64, CI 1.18-2.28). Furthermore, nonusers compared to LLM users were less likely to agree that LLMs will improve functional roles (63%, 140/221 vs 75%, 469/624; OR 0.60, CI 0.43-0.85). Free-text opinions highlighted concerns regarding autonomy, outperformance, and reduced demand for care. Respondents also predicted changes to human interactions, including fewer but higher quality interactions and a change in consumer needs as LLMs become more common, which would require provider adaptation.

**Conclusions:**

Despite the reported benefits of LLMs, nonusers—primarily health care workers, older individuals, and females—appeared more hesitant to adopt these tools. These findings underscore the need for targeted education and support to address adoption barriers and ensure the successful integration of LLMs in health care. Anticipated role changes, evolving human interactions, and the risk of the digital divide further emphasize the need for careful implementation and ongoing evaluation of LLMs in health care to ensure equity and sustainability.

## Introduction

The health care sector faces substantial workforce challenges driven by aging populations, increased chronic disease prevalence, and growing health care demands [1,2]. Consequently, technology is increasingly adopted to improve the quality and efficiency of care while alleviating workforce burdens [3,4]. Among the many technological developments, artificial intelligence (AI) has garnered much interest in the health care sector due to its ability to analyze, interpret, and generate actionable insights from large volumes of complex health data, transforming how care is delivered and managed [3]. Most recently, large language models (LLMs) have been changing how we use text, numeral, audio, and visual data, which will have widespread implications in the health care sector [5-9].

LLMs, a specific type of conversational AI, are trained to understand and generate human-like text [7]. While the foundational concepts of LLMs have been around for some time, there has been a significant leap in development in recent years. General purpose models like OpenAI’s ChatGPT, Google Gemini Ultra, Meta Llama 3, and Anthropic Claude 3, as well as domain-specific LLMs (BlueBERT, Copilot, and Med-PaLM) [7,9,10] are at the forefront of LLM capabilities, and interest in how these tools can benefit health care is only growing [7,9]. For example, LLMs to draft responses to patient messages, create structured medical notes of physician-patient interactions, provide clinical decision support, screen and enroll research participants, and enhance learning and education are only a few areas where LLMs are being applied in health care [7,9,11-17].

While the promise of LLMs in health care is substantial, it is also essential to understand their utility and challenges in real-world scenarios. For instance, early LLMs tended to “hallucinate” or provide inaccurate information, which would represent a significant risk if adopted in the health care context [16,18]. Newer innovations, including the creation of domain-specific LLMs, the incorporation of human feedback on responses, and the application of real-time domain-specific knowledge to enhance the performance of general LLMs (nonmedically trained), are now helping to minimize this issue [7,19,20]. Another limitation is a lack of clarity on how LLMs generate responses. The ongoing development of explainable AI models will aid in overcoming this problem by providing transparency in the decision-making process [21-23]. Finally, the need for adequate safeguards and regulation is another common concern. As regulations evolve, such as the liability rules outlined by the European Commission, ethical safeguards and clear frameworks for implementing LLMs into practice will be established [24].

Given the challenges with LLMs, the perspectives of health care workers, who represent both users and stakeholders who will influence and be influenced by others using these tools (eg, patients and other support functions), become particularly important for successful deployment. Moreover, understanding the influence of different backgrounds is also key. For instance, it is known that age and gender can significantly influence how individuals perceive and interact with technology [25-27]. Older individuals can be more skeptical and less trusting of technologies, including AI [26]. Conversely, younger generations may be more accustomed to digital technologies and report more positive views of technology [26]. Likewise, males are more likely to use digital applications, reporting more favorable views and being more confident technology users [27]. Frameworks like the Unified Theory of Acceptance and Use of Technology highlight the importance of such factors in influencing technology use [25].

By examining health care providers' perceptions of LLMs, we aim to identify gaps between user expectations and reality, and how this might vary by profile. This understanding is key for adoption, ensuring that these models align with users’ needs and ultimately improve patient care and health care efficacy. The purpose of this study is to explore professional views on LLM use in health care to inform future deployments. Specifically, we aim to (1) investigate users’ uptake, perceptions, and experiences regarding LLMs in health care and (2) contextualize survey responses by demographics and professional profiles.

## Methods

### Overview

This study is reported according to the Consensus-Based Checklist for Reporting of Survey Studies (Multimedia Appendix 1) [28]. In this cross-sectional study, we developed and administered an electronic survey to elicit stakeholder perspectives and experiences of LLMs and their potential impact on functional roles. A visual overview of the study flow is reported in Multimedia Appendix 2.

### Setting and Study Population

We adopted a convenience sampling approach. Recruitment occurred through a combination of electronic email blasts (distributed through the National University Health System email lists) and word of mouth (adverts sent to department leads and a prompt included in the survey invitation encouraging sharing among peers). The survey was aimed at health care professionals, health care professional students, or academics in related fields, regardless of institution. The survey was first launched on July 13, 2023 and a reminder email blast was sent on August 21, 2023. The survey eventually closed on October 16, 2023. Eligibility criteria are mentioned in Textbox 1.

**Eligibility criteria.**

**Inclusion criteria**

- Any health care providers (any profession or setting), students training in any health care discipline, or academics from health-related fields.
- Adults aged 18 years or older.
- Awareness of large language models.

**Exclusion criteria**

- Participants from fields unrelated to health care.
- No awareness of large language models such as ChatGPT.

We did not exclude those who had yet to use an LLM, so long as they had heard of them.

For those yet to use an LLM, survey questions relating to user experience were omitted.

### Survey Design

We developed the survey iteratively. First, JS and SYT reviewed the literature to identify common themes and issues related to the research questions. A draft survey was then shared with the wider project team to brainstorm new topics not otherwise captured and to refine questions. Finally, we assessed the content validity of the survey by circulating the survey with colleagues not within the project team (ie, health care providers and health care researchers) before launch. Feedback was used to improve the clarity of the survey before launch.

The final survey comprised 4 domains: demographics (6 questions), user experiences of LLMs (8 questions), motivations for using LLMs (6 questions), and perceived impact on their functional role (4 questions). The questionnaire took about 10 minutes to complete, and responses were anonymous. The survey consisted of multiple-choice questions, Likert scales, binary responses (ie, yes or no), and open-ended questions. The survey was launched in English on Qualtrics XM, a web-based survey platform. Although Singapore is a multilingual country, English is the default business language. Finally, a single question was included at the start of the survey: “Have you heard of LLMs such as ChatGPT before?” to assess eligibility. A copy of the survey is included in Multimedia Appendix 3.

### Data Analysis

Analyses were performed in STATA (version 15.0; StataCorp) and Microsoft 365 Excel (version 16.78). Summary statistics are presented as means with SD or proportions. Demographics for nonusers and early-LLM adopters were compared using a *t* test or chi-square test as appropriate. Significance was set at *P*<.05.

For categorical questions, the proportion of those who strongly or partially agreed with a question was calculated. Proportions were calculated for the whole sample and by specific demographical traits (sex, age group (18-29 years, 30-40 years, 41-50 years, and 50 years), ethnic group (Chinese, Malay, Indian, and Other), residency status (Singaporean or permanent resident, or employment pass or other pass holder), highest education level (0-level or diploma or higher degree) and job category (health care provider or student or health care support function)). The calculated proportions were then used to generate a heat map in Excel to visualize the data.

Ordinal regression (for Likert scale questions) or logistic regression (for binary questions) were used to explore whether participant characteristics were predictive of positive survey responses. Independent variables included: sex, age group, ethnic group, residency status, highest education level, job category, and whether they were an LLM user or not. For logistic regression, the dependent variable was binary coded (positive vs nonpositive). For ordinal regression, the dependent variable was collapsed into 3 categories (positive, neutral, or nonpositive). Odds ratios (ORs) with 95% CIs were computed for each predictor to interpret the strength and direction of associations. Robust SE was used in each analysis. Models were assessed for goodness-of-fit using the Hosmer-Lemeshow test for logistic regression and pseudo-R² metrics for ordinal regression.

For the open-ended survey questions, we performed a content analysis [29]. First, the free text was checked and corrected for spelling mistakes, slang, and abbreviations. Second, a subset of the data was analyzed and used to generate an initial set of themes. The themes were generated by one researcher and verified by the project team. Third, the themes were applied to the full dataset, further modifications were made to the codebook if required through discussion among the study team. Finally, the coded data was then checked by a researcher to verify the appropriateness and accuracy of the coding.

### Ethical Considerations

The study was reviewed and approved by the National University of Singapore Ethical Review Board (NUS-IRB-2023-211). The web-based survey was self-administered, with the Participant Information Sheet and Consent Form shown at the start of the web-based survey. Participants could only proceed after indicating their consent to participate in this research. Data were anonymous and analyzed at the aggregate level. Participants were not individually compensated but had the chance to enter a lucky draw, with fifty Singapore $20 (US $15) prizes awarded at random.

## Results

### Overview

We received a total of 1083 responses. After data cleaning 940 completed responses remained. A further 95 respondents were excluded from the dataset as they had never heard of LLMs such as ChatGPT; their characteristics are reported in Multimedia Appendix 4. Participant characteristics for the final analysis sample (n=845) are reported in Table 1. Of the 845 respondents, 221 had yet to use an LLM. Nonusers were more likely to be older, female, employment pass holders, and health care providers (*P*<.01).

**Table 1:** Survey respondent characteristics, overall and by LLM^a^ users and non-LLM users

Characteristic			All survey respondents N=845		Non-LLM users N=221		LLM users N=624	
**Age (years), mean (SD)**			35.49 (9.43)		37.72 (10.03)		34.70 (9.09)^b^	
**Male, n (%)**			240 (28)		45 (21)		195 (31)^c^	
**Ethnicity, n (%)**								
	Chinese	707 (84)		174 (79)		533 (85)		
	Malay	43 (5)		15 (6)		28 (5)		
	Indian	51 (6)		14 (6)		37 (6)		
	Other	44 (5)		18 (9)		26 (4)		
**Residency status, n (%)**								
	Singaporean	783 (93)		195 (88)		588 (94)^c^		
	Employment pass	48 (6)		23 (11)		26 (4)		
	Other (ie, student)	14 (1)		3 (1)		10 (2)		
**Education, n (%)**								
	O-level or N-level	8 (1)		2 (1)		6 (1)		
	Diploma or A-level	160 (19)		44 (20)		116 (19)		
	Higher degree	677 (80)		175 (79)		502 (80)		
**Role or occupation, n (%)**								
	Health care provider^d^ or student	552 (65)		164 (74)		388 (62)^b^		
	Health care administration^e^ or support functions	293 (35)		56 (26)		236 (38)		

^a^LLM: large language model.

^b^*P*<.001.

^c^*P*<.01.

^d^Doctors, nurses, allied health, dentists, and pharmacy staff.

^e^Operations, IT, finance, research, and corporate roles related to health care.

### Survey Responses

Results for the regression analyses are reported in Table 2. The 2 most frequent and significantly associated factors for positive survey responses were being a non-LLM user (associated with 7 questions) and increasing age (associated with 4 questions). The subsequent results are presented in three sections according to the three survey domains: (1) user experience of LLMs, (2) motivations for using LLMs, and (3) perceived impact on functional roles.

**Table 2:** Statistically significant participant characteristics associated with positive survey responses.

Survey domain and question		Significantly associated characteristic	Odds ratio (95% CI)	*P* values
**User experiences**				
	Positive user experience	Male	1.62 (1.06-2.48)	.02
	LLMs^a^ are accurate	None	—^b^	—
	LLMs are useful	Increasing age	0.98 (0.96-0.99)	.03
	Confident using LLMs	Non-LLM user	0.31 (0.22-0.44)	<.001
	Recommends LLMs	Non-LLM user	0.10 (0.07-0.15)	<.001
**Motivations for use**				
	LLMs are not overhyped	Increasing age	0.97 (0.96-0.98)	.001
	LLMs are not overhyped	Non-LLM user	0.67 (0.50-0.90)	<.01
	No social expectation to use LLMs	Increasing age	0.96 (0.95-0.97)	<.001
	No social expectation to use LLMs	Singaporean or permanent resident	1.66 (1.04-2.64)	.03
	LLMs address an unmet need	Male	1.64 (1.18-2.28)	.003
	LLMs address an unmet need	Non-LLM user	0.48 (0.35-0.65)	<.001
**Perceived impact on functional role**				
	LLMs will improve functional roles	Chinese	2.52 (1.06-5.97)	.03
	LLMs will improve functional roles	Malay	3.17 (1.07-9.36)	.03
	LLMs will improve functional roles	Non-LLM user	0.60 (0.43-0.85)	<.01
	LLMs will impact human interactions	Increasing age	1.02 (1.00-1.03)	<.01
	LLMs are not a threat to functional roles	Higher degree holder	1.84 (1.26-2.70)	.002
	LLMs are not a threat to functional roles	Non-LLM user	0.65 (0.46-0.91)	.01
	LLMs should be used in health care	Higher degree holder	1.80 (1.09-2.98)	.02
	LLMs should be used in health care	Non-LLM user	0.62 (0.38-0.98)	.04

^a^LLMs: large language models.

^b^Not applicable.

### User Experiences of LLMs

**Figure 1:**
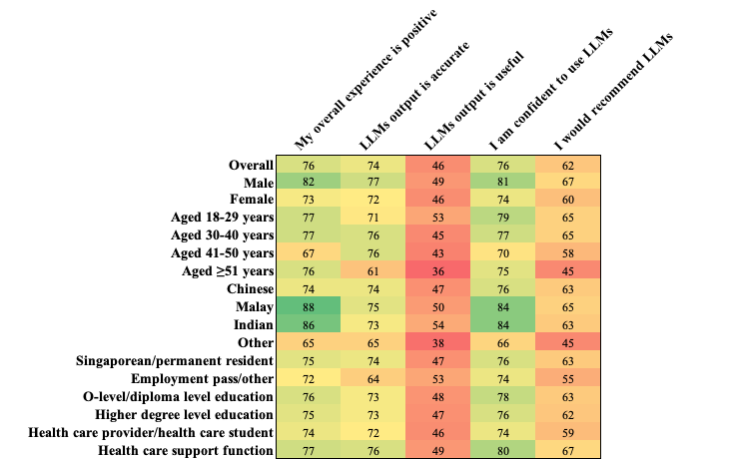
A heat map of question responses (percentage agreement) on user experience, overall, and by individual demographic groups. LLM: Large Language Model.

Roughly half of users reported rarely using LLMs (323/624, 52%), and 40% (249/624) reported weekly use. More than 70% rated the overall experience (470/624), the perceived accuracy (548/625), and their confidence in using LLMs highly (644/845), and 62% (524/845) would recommend LLMs to others. Conversely, less than half (294/624, 46%) agreed that the content generated by the LLM was useful (Figure 1; dark green indicates strongest agreement, and dark red indicates lowest agreement).

**Figure 2:**
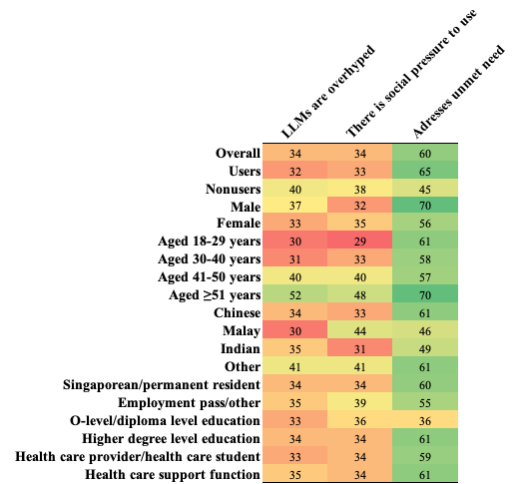
A heat map of question responses (percentage agreement) on motivations for using large language models, overall, and by individual demographic groups. LLM: Large Language Model.

Regression analysis showed that the experience with LLMs is more likely to be positive if the user is male, and increasing age was associated with a reduced likelihood of reporting LLM output as useful (Table 2). Non-LLM users were less likely to report feeling confident to use LLMs and less likely to recommend them to others. In Figure 2, dark green indicates the strongest agreement and dark red indicates the lowest agreement.

### Motivations for Using LLMs

Approximately half of the respondents used LLMs for personal and work reasons (333/624), and users (407/624, 65%) were more likely to report LLMs addressed an unmet need compared to nonusers (99/221, 45%; Figure 2). Around a third of users and nonusers agreed that external factors motivated decisions to use LLMs (ie, social pressure or hype).

Regression analysis (Table 2) found that younger adults and LLM users were more likely to disagree that LLMs are overhyped, and younger adults and Singaporeans or permanent residents were more likely to report there are no social expectations to use LLMs. Furthermore, males were more likely to report that LLMs addressed an unmet need, but non-LLM users reported the opposite.

When asked about the motivation for using LLMs, the top 5 reasons were speed, productivity, convenience, curiosity, and personalized responses. In terms of specific tasks, roughly two-thirds of respondents reported using LLMs for ideation (391/624, 63%), answering general questions (381/624, 61%), and writing (359/624, 57%). Other uses included entertainment (204/624, 33%), answering medical questions (137/624, 22%), data analysis (109/624, 17%), social interaction (100/624,16%), and literature reviews (95/624, 15%).

When asked why the nonusers had not used LLMs yet, content analysis of free text revealed 5 topics. First, there was a (1) lack of trust or skepticism among users regarding the reliability of AI-generated content, coupled with concerns about data security and the software’s credibility. In addition, some users perceived (2) no immediate need or relevance for AI tools, relying on alternative sources for information, such as established search engines (eg, Google). Others (3) lacked awareness of LLMs and their potential applications, and some did not have (4) access or an opportunity to try LLMs, citing financial hurdles, lack of institutional investment, or lack of time as reasons. Finally, some reported a lack of (5) technology literacy, which prevented them from trying LLMs.

### Perceived Impact on Functional Role

**Figure 3:**
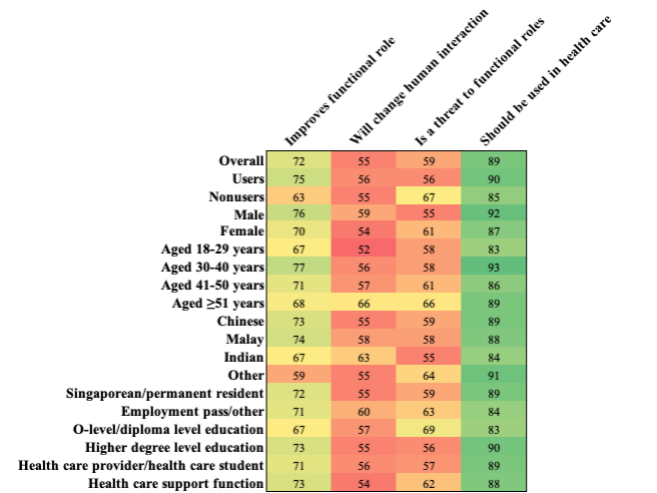
A heat map of question responses (percentage agreement) on the perceived impact of large language models on functional roles, overall and by individual demographic groups.

Regardless of profile, agreement that LLMs should be used in health care was >80% (749/845; Figure 3). Regression analyses (Table 2) revealed that non-LLM users were statistically significantly less likely to agree that LLMs will improve functional roles, LLMs are not a threat to functional roles, and LLMs should be used in health care. Other factors significantly associated with survey responses in this domain were ethnicity, education level, and age. In Figure 3, dark green indicates the strongest agreement and dark red indicates the lowest agreement.

When survey respondents were asked about their views on the perceived impact of LLMs on functional roles, content analysis of free text revealed three main topics. First, there is (1) apprehension that LLMs could compete for tasks traditionally performed by health care workers. For example, increasingly accurate diagnostics and scan interpretations or the automated generation of treatment plans could threaten doctors' autonomy. Second, the ability of LLMs to synthesize and interpret vast amounts of information could also lead to (2) greater accuracy and efficiency in practice. Consequently, LLMs could outpace doctors' ability to stay current. Third, as (3) LLMs improve the accessibility of medical information, there may be a reduced demand for consultations and a devaluing of domain expertise.

Despite these concerns, some health care workers believe that the integration of LLMs will not be immediate, given the technology's current limitations, particularly its reliability and accuracy. There is also recognition that LLMs could create new opportunities within the sector, though uncertainty remains about the extent of their impact. Importantly, participants emphasized the need for human oversight to ensure the accuracy and relevance of LLM-generated content, highlighting safety concerns such as the potential for scams, data breaches, and misinformation.

### The Impact of LLMs on Human Interactions

**Figure 4:**
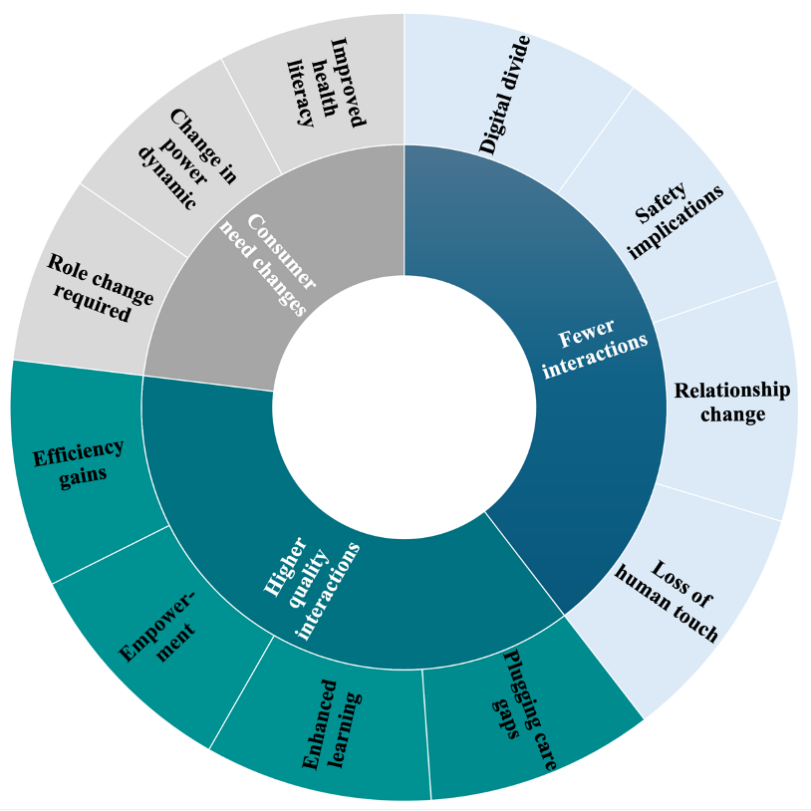
Qualitative themes (inner circle) and subthemes (outer circle) on the perceived impact of large language models on human interactions from the free text survey data.

When asked about how LLMs might impact human interactions, 3 topics emerged from the analysis of free text responses: less interactions, higher quality interactions, and a change in consumer needs (Figure 4).

### Topic 1: Fewer Interactions

Participants reflected that introducing LLMs would likely reduce the volume of human interactions. Participants expressed concerns about a loss of human touch or empathic interactions, as LLMs may not fully replicate the nuances of human interaction. Furthermore, fewer human interactions may negatively impact the patient and provider relationship (or teacher and student), raising concerns about the erosion of rapport and trust. The safety of reduced interactions was another concern, particularly for patients whose issues may only be fully understood with in-person discussion, creating missed opportunities to engage and address care needs. Moreover, certain aspects of human interaction were viewed as irreplaceable, highlighting the indispensable need for the human touch in health care settings.

### Topic 2: Higher-Quality Interactions

Respondents noted that LLMs can potentially improve the quality of human interactions. For example, integrating AI to handle simpler, less complex tasks could free up time for professionals to focus on more critical responsibilities where human interaction is essential. In turn, this was seen as a means to enhance efficiency and effectiveness. Participants expressed optimism about the empowerment derived from having greater accessibility to knowledge, leading to more informed interactions and improved learning experiences for practitioners, patients, and students. Furthermore, respondents theorized that using AI could help address care gaps in situations where patients have limited access to care.

### Topic 3: Change in Consumer Needs

Participants highlighted that LLMs would likely change consumer knowledge and attitudes (ie, patients and students), leading to shifts in their needs and the relationship dynamics. For instance, as patients become more health-literate, their inquiries and demands will evolve. Empowered consumers, informed by LLMs, may also disrupt traditional power dynamics (ie, patient and provider, teacher and student) where professionals are the traditional knowledge keepers. These consumer changes would likely necessitate acquiring new interpersonal skills to manage the demands of a more health-literate population and managing misinformed consumers. Conversely, LLMs may also lead to a digital divide, with less technologically savvy individuals being left behind, exacerbating existing disparities in technology use and access to health care and education.

## Discussion

### Principal Findings

We conducted a cross-sectional survey to gather stakeholder perspectives and experiences of LLMs and their potential impact on functional roles in health care. We received over 800 analyzable responses from health care providers, support staff, health care students, and academics in related fields. Among the respondents, nonusers, were predominantly health care workers, older individuals, and females. Users primarily adopted LLMs for speed, convenience, and productivity. While the overall user experience was generally positive, approximately half reported that the content generated was not useful. In contrast, nearly 90% of respondents felt LLMs should be used in health care. Nonusers were less likely to recognize LLMs as addressing unmet needs or improving functional roles. Free-text opinions highlighted concerns regarding autonomy, outperformance, and reduced demand. Furthermore, respondents felt that human interactions would likely change, expecting fewer but higher quality exchanges as well as shifts in consumer attitudes and needs, which would require provider adaptation.

Most respondents rated their overall experience and confidence in using LLMs highly. LLMs have garnered significant popularity due to their user-friendly interface, accessibility, and ability to generate human-like output promptly [30]. Motivation for using LLMs was predominantly driven by the desire for speed, convenience, and productivity, with about 70% of respondents citing these factors. This finding aligns with current opinion on using LLMs in health care [5,19,31,32]. However, less than half found the output generated useful, indicating a gap between the positive user experience and the perceived utility of LLM-generated content. This disconnect may be explained by known issues when using LLMs, such as hallucinations, poor accuracy of responses, a lack of explainability, and suboptimal user prompting [33-35]. Recent advancements in LLMs are addressing these key limitations, such as fine-tuning LLMs using domain-specific datasets to improve accuracy and reliability, new training methodologies (eg, reinforcement learning with human feedback) to align LLM-generated outputs more closely with expert-validated data [18], real-time detection and mitigation of hallucinations, and ongoing work on explainable AI models to ensure transparency and build trust [19,21-23,36]. Finally, as users become more familiar with LLMs, their awareness and skill at tailoring prompts to generate more accurate responses will improve.

There was a mixture of views on the perceived impact of LLMs on functional roles, but most felt that LLMs should be used in health care. This sentiment is echoed in similar surveys, which report favorable views on LLM use in health care, emphasizing LLMs as copilots rather than role replacers [31,32,37]. Adopting LLMs as copilots would mitigate the risk of using inaccurate information by maintaining human oversight. As with the perceived impact on functional roles, respondents also had mixed opinions of the impact of LLMs on professional interactions. Some foresee reduced human interactions, eroding empathy, and negatively impacting the patient-provider relationship. Furthermore, LLM use in health care may exacerbate the digital divide, as those without access or less technologically savvy individuals are left behind [38].

These perspectives highlight the complexity and uncertainty surrounding the adoption of LLMs in health care settings, emphasizing the importance of continued research and evaluation. Respondents reported a mix of positive and negative implications of LLMs, and these views varied by demographic profile. In particular, non-LLM users and age were associated with responses in many survey domains. Previous research has shown that age, sex, and experience influence technology adoption [25]. Increasing age is linked to poorer technology understanding and engagement [26,39]. Women are more hesitant to adopt technology due to an underrepresentation in AI development roles and a lower proportion of women studying STEM subjects, hindering their exposure to and interest in technology [27,40,41]. Finally, previous experience can significantly impact future intentions to use technology [25]. By taking into account the factors that influence perception and adoption, and improving education on LLM technologies, development and implementation can be enabled.

Notwithstanding the perceived benefits of LLMs in health care, further research is needed to evaluate the actual impact of implemented LLM technology. Evaluation is needed to establish whether promises of improved efficiency, greater accuracy, and improved patient care are true or if such technology introduces new challenges. As LLM technology becomes more commonplace within the health care sector clinicians will also need training. Training will help to optimize LLM usage (ie, prompt engineering), and how to handle patients who become active users of such technology. Finally, it is important that inequalities are not exacerbated or introduced through the introduction of LLMs into health care. Supporting slow adopters, including women and older adults, is also critical for successful implementation.

### Limitations

There are several strengths and limitations to consider when interpreting the results. As a cross-sectional study, our study may be prone to issues with bias due to the study design. For instance, the convenience sampling approach may have led to a sample that is not fully representative of the broader health care workforce. We attempted to address this by maximizing our sampling approach and disseminating the survey through multiple routes. The limitation of an electronic survey format may have also resulted in a biased sample agreeing to participate, for instance, those who are more technology literate. Finally, participants may have provided socially acceptable responses, particularly as respondents had a chance to win an incentive. We attempted to mitigate this risk by making the survey anonymous. Future studies should attempt to use more diverse recruitment strategies to reach underrepresented groups.

### Conclusions

LLMs can be valuable tools that can enhance and augment health care roles. However, health care inherently relies on nuanced decision-making, patient trust, physical interaction, and empathy—qualities that LLMs cannot replace. Nonusers, predominantly health care workers, older individuals, and females in our study, remain hesitant to adopt these tools, underscoring the need for targeted education and support to overcome barriers. Anticipated role changes, evolving human interactions, and the risk of the digital divide further highlight the importance of careful integration and ongoing evaluation of LLMs to ensure equity and sustainability in health care.

## Data Availability

The data are available upon reasonable request to the corresponding author.

## Appendix

Multimedia Appendix 1 Consensus-Based Checklist for Reporting of Survey Studies.

Multimedia Appendix 2 Study flow.

Multimedia Appendix 3 Survey template.

Multimedia Appendix 4 Characteristics of excluded participants.

## Abbreviations

AI: artificial intelligence
LLM: large language model
OR: odds ratio
