# Supplementary figures and images for: Perspectives and Experiences With Large Language Models in Health Care: Survey Study
Source: J Med Internet Res. 2025 May 1;27:e67383. doi: 10.2196/67383 (PMC12082058; doi:10.2196/67383)

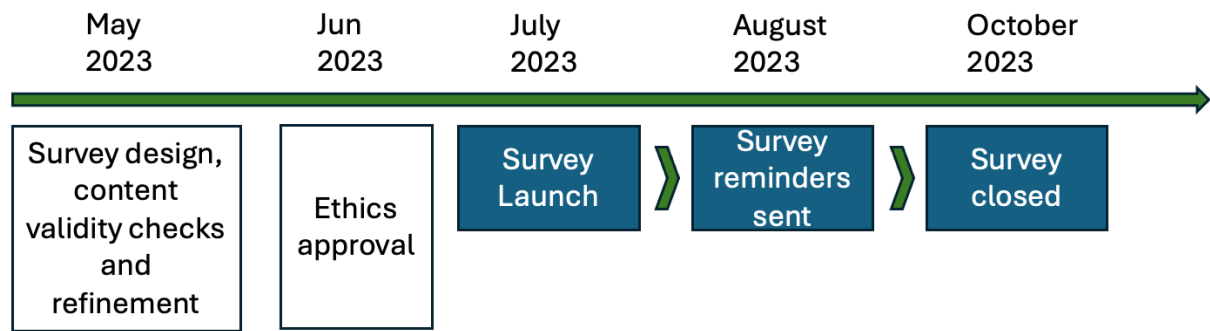

Supplement: Multimedia Appendix 2 [file jmir_v27i1e67383_app2.pdf]
