# Supplementary material for: Perspectives and Experiences With Large Language Models in Health Care: Survey Study
Source: J Med Internet Res. 2025 May 1;27:e67383. doi: 10.2196/67383 (PMC12082058; doi:10.2196/67383)
Supplement: Multimedia Appendix 3 [file jmir_v27i1e67383_app3.pdf]

Exploring the role of LLMs in healthcare

Section 1. Demographics

1. Age_______
2. Gender:
   [ ] Male [ ] Female
3. Ethnicity:
   [ ] Chinese
   [ ] Malay
   [ ] Indian
   [ ] Others

4. Residency status:
   [ ] Singaporean/PR [ ] SPass, EP holder [ ] Other_______

5. Highest qualification
   [ ] Diploma
   [ ] Bachelors
   [ ] Masters
   [ ] PhD
   [ ] MBBS/MD
   [ ] Others_______

6. Role/Occupation:
   [ ] Doctor
   [ ] Nurse
   [ ] Pharmacist
   [ ] Allied Health
   [ ] Medical student (doctor)
   [ ] Nursing student
   [ ] Pharmacy student
   [ ] Allied health student
   [ ] Healthcare administration
   [ ] Academic
   [ ] Others_______

7. Number of years of clinical or professional practice (if applicable)_______

8. Practising institution:

[ ] Private healthcare   [ ] Public healthcare   [ ] Academia   [ ] Other_______

Section 2. Knowledge and experience in using LLMs

9. Have you heard of LLMs such as ChatGPT?

   [ ] Yes   [ ] No

10. Have you used LLMs like ChatGPT? If not, why? _______

    [ ] Yes   [ ] No

11. Do you feel confident that you can use LLMs effectively?

    [ ] Yes   [ ] No

12. Did you use it for personal reasons or for work?

    [ ] Personal use   [ ] Work-related   [ ] Both   [ ] N/A

13. What did you use it for? [you may select multiple options]

    [ ] Writing
    [ ] Literature reviews
    [ ] Answering medical questions
    [ ] Answering general questions
    [ ] Exams or coursework
    [ ] Data analysis
    [ ] Ideation/Brainstorming
    [ ] Social interaction
    [ ] Entertainment
    [ ] Others_______
    [ ] N/A

14. How often do you use it?

    [ ] Never   [ ] Rarely (monthly)   [ ] Occasionally (weekly)   [ ] Frequently (every day)

15. Overall, how would you rate your experience of using LLMs?

[ ] Very Poor   [ ] Poor   [ ] Neutral   [ ] Good   [ ] Very Good

16. Overall, do you perceive the responses to be accurate and reliable?

[ ] Very inaccurate and unreliable   [ ] Somewhat inaccurate and unreliable   [ ] Neutral   [ ] Somewhat accurate and reliable   [ ] Very accurate and reliable

17. Overall, do you find the responses useful?

[ ] Very useless   [ ] Somewhat useless   [ ] Neutral   [ ] Somewhat useful   [ ] Very useful

Section 3. Impacts of LLMs to your functional roles

18. Do you think LLMs will improve your current functional role?

[ ] Yes   [ ] No

If yes, how_______

19. Do you think LLMs will ever pose any threat to your job security:

[ ] in the next 5 years   [ ] in the next 10 years   [ ] beyond 10 years   [ ] Never

If yes, why_______

20. Do you think LLMs should be used in the healthcare setting?

[ ] Yes   [ ] No

If yes or no, why_______

21. Do you think LLMs will change your interactions with patients and/or students?

[ ] Yes   [ ] No

If yes or no, why_______

Section 4. Motivations for using LLMs

22. What motivates you to use LLMs like ChatGPT?

    [ ] Convenience/accessibility

    [ ] Speed of answer

    [ ] To increase productivity

    [ ] Personalised responses

    [ ] Curiosity/novelty

    [ ] Anonymity

    [ ] It's better at performing a task than me

    [ ] Nothing

    [ ] Others_______

23. Do you think LLMs are overhyped?

    [ ] Yes [ ] No

24. Do you think LLMS address an unmet need?

    [ ] Yes [ ] No

25. Do you feel there is a social expectation that you should use LLMs?

    [ ] Yes [ ] No

26. Is there anything you would not use LLMs for? _______

27. Have you recommended LLMs to others?

    [ ] Yes [ ] No

If yes, for what reason_______

Section 5. Concluding comments

28. Do you have any other comments on LLMs you would like to share?

____
