# Supplementary material for: Perspectives and Experiences With Large Language Models in Health Care: Survey Study
Source: J Med Internet Res. 2025 May 1;27:e67383. doi: 10.2196/67383 (PMC12082058; doi:10.2196/67383)
Supplement: Multimedia Appendix 4 [file jmir_v27i1e67383_app4.pdf]

Characteristics of those not aware of LLMs

Characteristic	All survey respondents N=95
Mean age, years (SD)	39.29 (9.76)
Male, n (%)	5 (5)
Ethnicity, n (%)	
Chinese	52 (55)
Malay	17 (18)
Indian	17 (18)
Other	9 (9)
Residency status	
Singaporean	83 (87)
Employment pass	12 (13)
Education, n (%)	
O-level/N-level	7 (7)
Diploma/A-level	36 (38)
Higher degree	49 (52)
Not reported	3 (3)
Role/Occupation	
Healthcare provider	71 (75)
Healthcare administration/support functions	20 (21)
Healthcare student	4 (4)

Abbreviations: *Standard Deviation (SD)*

Healthcare provider: *Doctors, nurses, allied health, dentist, pharmacy staff;* ***Healthcare administration/support functions:*** *Operations, IT, finance, research, corporate roles related to healthcare*
